# Identification of The Canidae Iron Regulatory Hormone Hepcidin

**DOI:** 10.1038/s41598-019-55009-w

**Published:** 2019-12-18

**Authors:** Martin K. Mead, Melissa Claus, Edward Litton, Lisa Smart, Anthea Raisis, Gabriele Rossi, Robert D. Trengove, Joel P. A. Gummer

**Affiliations:** 1 School of Veterinary and Life Science, Murdoch University, Perth, WA Australia; 2 School of Veterinary Medicine, Murdoch University, Perth, WA Australia; 3 Intensive Care Unit, Fiona Stanley Hospital, Perth, WA Australia; 4 School of Medicine, The University of Western Australia, Perth, WA Australia; 5 Metabolomics Australia, Western Australia Node, Murdoch University, Perth, WA Australia; 6 Centre for Computational and Systems Medicine, Health Futures Institute, Murdoch University, Perth, WA Australia

**Keywords:** Chemical biology, Computational biology and bioinformatics, Molecular biology, Systems biology, Biomarkers

## Abstract

Hepcidins are an evolutionarily conserved class of liver-expressed peptide, from which the twenty-five amino acid hormone, hepcidin-25 (herein hepcidin), has gained significant notoriety as the master regulator of iron homeostasis in mammals. Hepcidin maintains iron homeostasis by controlling the dietary absorption of iron and the mechanisms of recycling cellular iron stores. With the physiological significance of this hormone well established, it has emerged as an informative biomarker. In a comparison of the genome, transcriptome and peptidome of *Canis lupis familiaris*, we reveal the size of the hepcidin peptide in the canine, previous reports of which were contradictory to the evolutionary conservation predicted by genome annotation. Here, measurement of the peptide by mass spectrometry, following isolation from greyhound blood serum, revealed an amino acid sequence and peptide mass, differing from all accounts to date, yet demonstrating perfect sequence identity to that of the greater Canidae lineage of the Carnivora. Importantly, in the greyhound, the measured hepcidin peptide showed a similar temporal pattern to total serum iron, consistent with our understanding of hepcidin regulating iron homeostasis, in agreement with human diagnostics, and providing added translational evidence of the measured peptide being the iron regulatory hormone of the Canidae.

## Introduction

The only input to the total iron content of mammalian systems, other than by clinical intervention is through the diet^1^. Because there exists no mechanism of controlled iron removal from mammals, iron regulation by physiological and biochemical recycling mechanisms, finely balanced by controlled dietary intake, each mediated by hepcidin-25 (HAMP; hepcidin antimicrobial peptide; herein hepcidin) are integral to maintaining the strict requirements of iron homeostasis^2^. By direct binding and resultant endocytosis of the iron exporter ferroportin, hepcidin regulates the release of iron into plasma from intestinal enterocytes, macrophages, hepatocytes, and placental cells^3–5^. Thus hepcidin maintains iron homeostasis by controlling the dietary absorption of iron and the mechanisms of recycling cellular iron stores. In mammals, low concentrations of systemic hepcidin promote iron absorption and are consistent with increased iron demand; such as is required for erythropoiesis^6^. Higher concentrations of hepcidin will deplete iron availability, by restricting absorption and promoting sequestration, including during infection^7^ or other inflammatory conditions^8^. Chronic deficiency in circulating hepcidin however is associated with the excessive absorption of dietary iron and clinical disorder of iron overload^9^. In excess, the negative regulation of ferroportin by hepcidin will reduce the transport of iron from the gut and sequester existing iron within cells^3^, reducing its physiological availability and which may lead to anaemias^10,11^.

The use of laboratory mice as models of iron metabolism has greatly advanced our understanding of hepcidin and iron regulatory processes more broadly^10,12,13^. It has been recognised that an availability of animal models with a more representative iron turnover to that of humans would also be advantageous for human translation^14^. More directly, hepcidin may yet prove useful in animal diagnostics^15^. It has been speculated that carnivores, such as dogs, can more readily absorb dietary heme than mice because they are evolutionarily carnivorous^16^, have a metabolic rate to body mass ratio more representative of humans^17^, and have a closer matched iron economy to humans; including of erythrocyte life-span^18–20^ and of total iron stores^15,21–23^. Further, whilst mice do not demonstrate equivalent toxicity of iron overload as humans^24^, iron is believed to contribute to the pathogenesis of diseases in dogs as it does in humans^22^.

**Figure 1:**
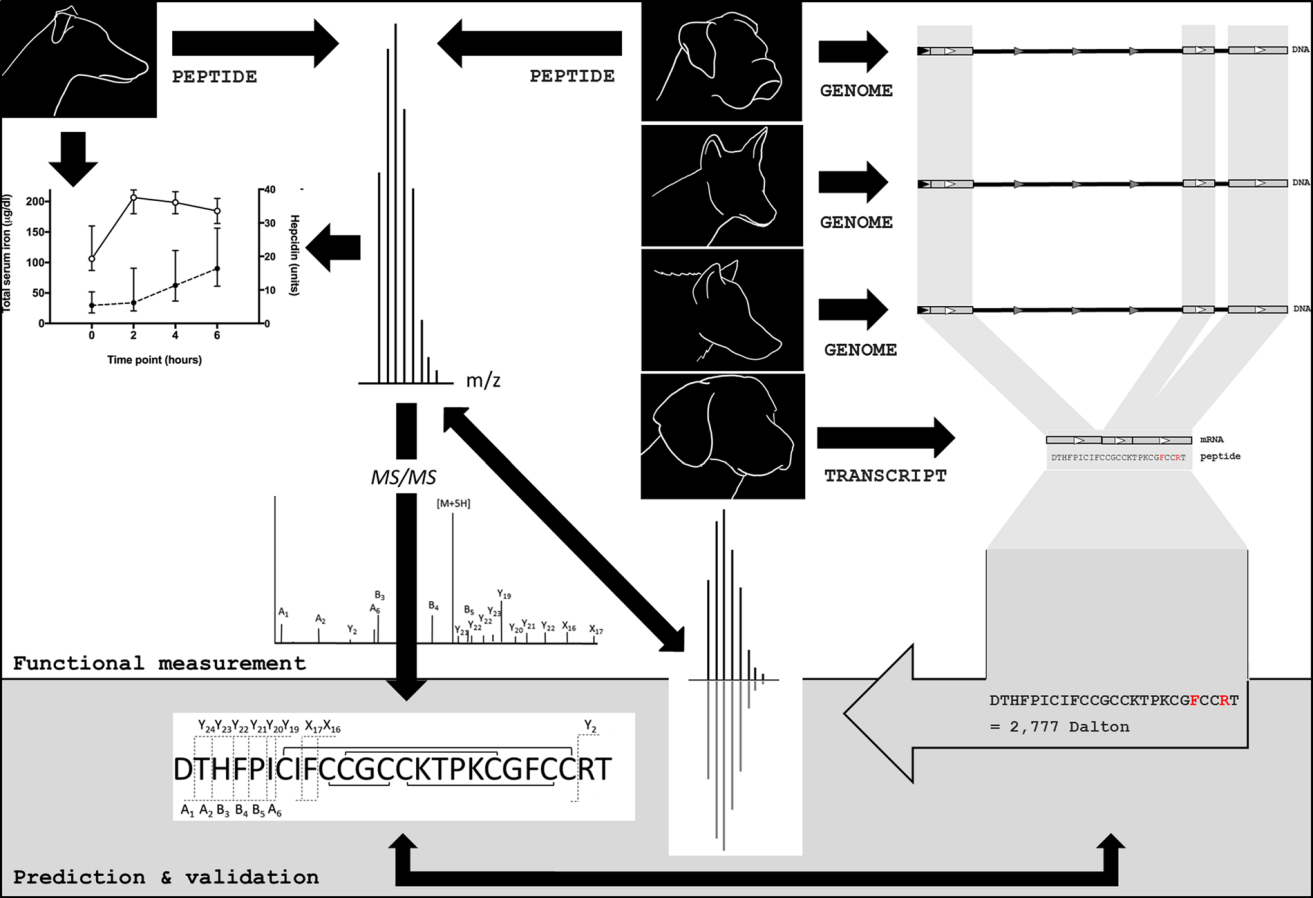
A peptidogenomics approach predicted amino acid sequence conservation between the translated hepcidin (*Hamp*) gene of *Canis lupis familiaris* (dog; boxer), *C. lupis lupis* (wolf), *C. lupis dingo* (dingo); and the translated cDNA of *C. lupis familiaris* (dog; beagle). The predictions were consistent with the measured mass and the derived amino acid sequence of the hepcidin peptide from *C. lupis familiaris* (dog; greyhound), as elucidated by mass spectrometry.

Until now, the direct measurement of the native hepcidin peptide in animals other than humans and mice has remained to be achieved. Our understanding of this integral homeostatic hormone has to date only been attained by mRNA expression of the hepcidin (*Hamp)* gene, or permitted by cross-reactivity of orthologous hepcidin peptides by ELISA^25^. Relative quantitation of this peptide has therefore only been indirectly determined in the systemic circulation of animals other than of humans and mice^25–28^. Mass spectrometry (MS) provides the capability to measure the ionised hepcidin peptide itself, and with efforts toward standardisation^29^ may yet prove more clinically informative than other analytical techniques of hepcidin measurement^30^. Further, by leveraging the specificity of MS, evolutionary differences in the amino acid sequence of the native peptide between species can be determined. Whilst measurement by MS has not been achieved in many animals, the translational potential of hepcidin for describing the iron regulatory phenotype of animal models^14^ and relevance of the peptide as a diagnostic marker in veterinary medicine may prove substantial. To this end, we used a peptidogenomics approach to measure and validate the identity of the endogenous dog hepcidin; combining an intact mass measurement of the peptide by MS and validation against a series of genome and mRNA sequence alignments of the Canidae Family (Fig. 1), and of the greater Carnivora Order. These data revealed the hepcidin amino acid sequence has until now been widely misreported.

## Results

### Measurement of the *C. lupis familiaris* hepcidin by mass spectrometry

**Figure 2:**
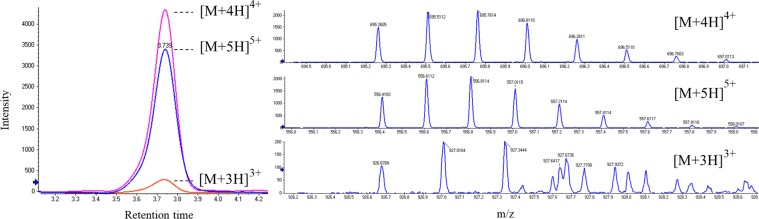
Overlayed extracted ion chromatograms of each observed charge state of the precursor hepcidin (left) as measured by LCqTOF-MS. The respective mass spectrum for ions representative of the [M + 5H]^5+^, [M + 4H]^4+^ and [M + 3H]^3+^ charge states, demonstrating the isotopic abundance are also pictured (right). Co-elution of another analyte(s) can be seen in the higher mass isotopes of the [M + 3H]^3+^ mass spectrum.

**Figure 3:**
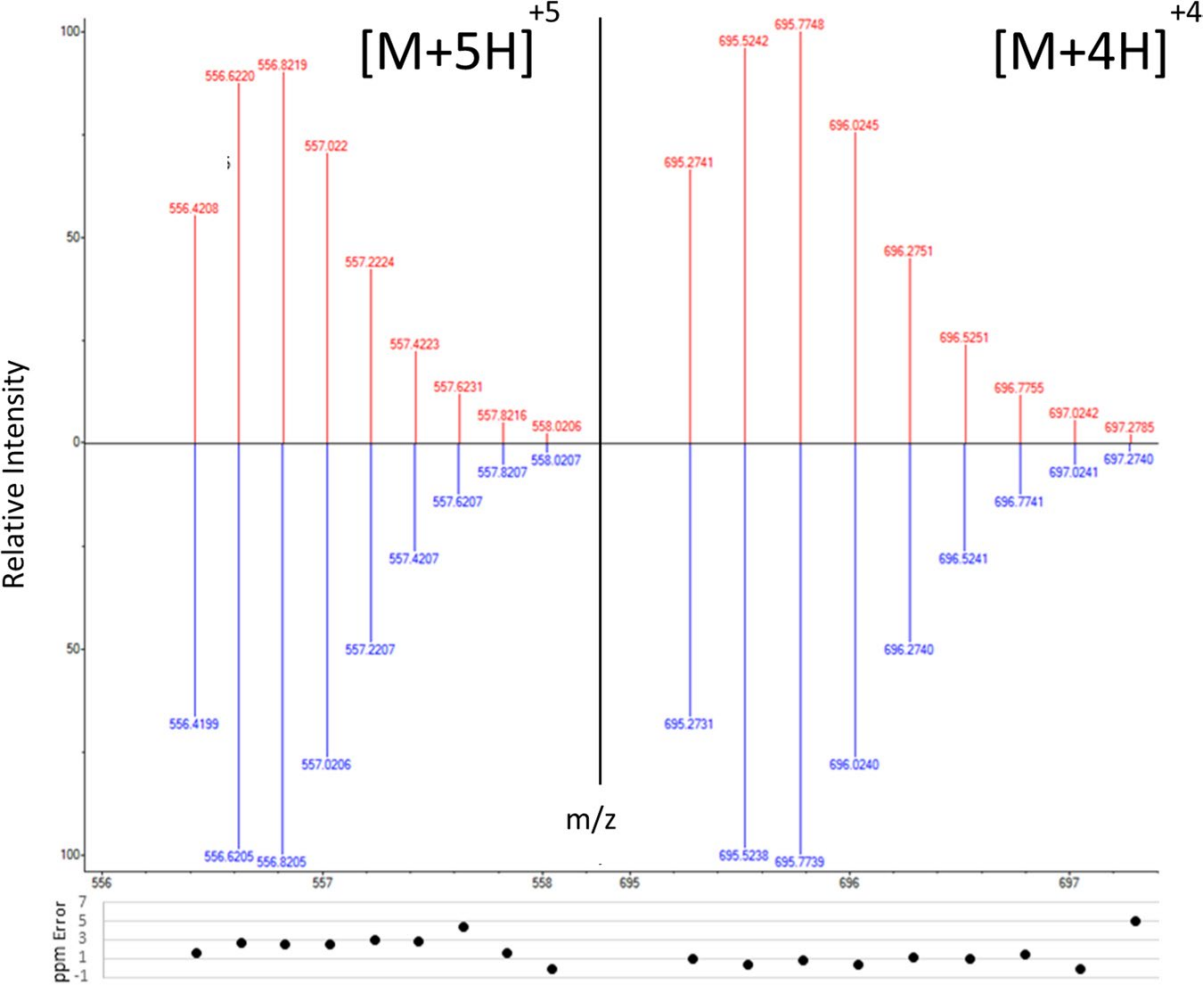
Top-down mass spectral comparison of the measured isotopes of the [M + 5H]^+5^ and [M + 4H]^+4^ charge state ions of the Canidae hepcidin peptide (top) and the predicted mass spectral pattern of the equivalent ions (down). The isotopic patterns show consistency between the theoretical pattern, and that of the ionised hepcidin peptide. The mass-to-charge ratios (m/z; x-axis) and relative intensities  (y-axis) are displayed. The calculated mass error (ppm) is shown.

**Figure 4:**
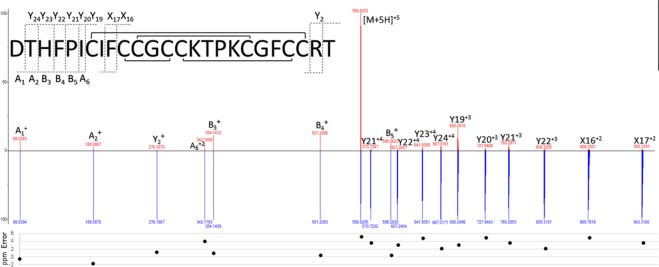
Top-down mass spectral comparison of the measured product ion mass spectrum (MS/MS) arising from the precursor ion [M + 5H]^+5^ of the *C. lupis familiaris* hepcidin used for amino acid sequence determination. The *de novo* amino acid sequence annotation (top) is compared to the predicted MS/MS spectra determined from the predicted translation of *Hamp* (DNA and mRNA) sequences (down). The mass-to-charge ratios (m/z; x-axis) and relative peak intensities (y-axis) are displayed. The calculated mass error (ppm) is shown.

The dog (*Canis lupis familiaris*) ortholog of hepcidin was uncovered using a top-down peptidomics approach. Peptides were isolated from greyhound (*C. lupis familiaris*) serum and measured intact using liquid chromatography (LC) coupled to accurate-mass MS. Of the peptides measured, none were consistent with the expected mass of the canine hepcidin as determined in previous literature accounts^26^. Three ions matching the charge state distribution (Fig. 2; [M + 5H]^+5^, m/z 556; [M + 4H]^+4^, m/z 695; and [M + 3H]^+3^, m/z 926) and isotopic abundance pattern of hepcidin when previously measured, or predicted to result from, electrospray ionisation (Figs. 3 and S1)^31,32^ were, however observed and determined to be of a 2,777 Dalton (Da) peptide. Due to the discrepancy between this result and previous literature accounts of the mass, the analytical methods were checked for amenability to a commercially available recombinant “Canine Hepcidin” standard (Peptides International, Kentucky USA). Analysis of the recombinant peptide revealed a mass of 2,715 Da, consistent with the originally reported cDNA translation^26^, however inconsistent with the hepcidin isolated from greyhound serum. The measurements were repeated using serum sourced from the *C. lupis familiaris* breeds; kelpie X (Cross), giant schnauzer, cavalier King Charles spaniel, Labrador retriever X and Great Dane. The same ions were measured in each breed and served to confirm that our determined putative hepcidin mass was likely that of all *C. lupis familiaris* (and interbreeds thereof) and not an anomaly of the greyhound, with breeding practices potentially selecting for the heightened iron demand of an elite athlete. Hepcidin and the proteoforms thereof are highly conserved through evolution such that sequence orthology of the peptide amongst species should yield confirmatory product ion spectra arising from fragmentation in the highly conserved amino terminus of the peptide (consistent with the human hepcidin peptide; S1)^31^. The putative canine hepcidin confirmed this hypothesis when dissociated in the collision cell of the MS, yielding the same characteristic ions of other 25 amino acid hepcidins (Figs. 4, S2). The resolved product ion spectrum was further used for *de novo* elucidation of the amino acid sequence identity (Figs. 4; S2) and reverse-translated for comparison to predictions of the *Canidae Hamp* conserved gene alignments.

### Peptidogenomics comparison against the *C. lupis familiaris* genome assembly

The *C. lupis familiaris* genomic region of *Hamp* predicted a translated amino acid sequence DTHFPICIFCCGCCKTPKCGFCCRT, with phenyalanine-‘F’ and arginine-‘R’ residues at the 21^st^ and 24^th^ positions, respectively. The F and R were noted to contradict previous accounts of leucine (L) and lysine (K) at these locations (Table 1). Reverse translation of the *de novo* peptide sequence and the *in silico* prediction of dog hepcidin mRNA (*Hamp*) translated from the *C. lupis familiaris* genome assembly, each provided a consistent amino acid sequence to our measurements (Fig. 1), yet one differing from the earlier reported cDNA sequence^33^; (GenBank: AY772532.1) and other database records^34^, (Table 1, S3). As part of the reference sequence collection of RefSeq the dog genome assembly (GCF_000002285.3) is a highly curated resource^35^; predictions from the dog genome identify a translation to F and R residues at the 21^st^ and 24^th^ peptide positions, respectively, and predicted the replacement of the earlier reported L and K/isoleucine-‘I’ residues designated to these locations in other accounts to date (Table 1). Translation of these data, which can be accounted for by a discrepancy in two incorrectly determined nucleotides of a cloned and sequenced RNA (cDNA)^26^, which when curated using the genomic sequence, anticipate a native mass of the dog hepcidin peptide to actually be 2,777 Dalton; consistent with the MS determination.

**Table 1:** Amino acid sequence translations of reported Canine hepcidins.

FUNCTIONAL MEASUREMENT	REPORTED TRANSLATION OF CANINE HEPCIDIN-25	SOURCE OF DATA
mRNA	DTHFPICIFCCGCCKTPKCG**L**CC**I**T	GenBank:AAT95397.1
mRNA	DTHFPICIFCCGCCKTPKCG**F**CC**K**T	GenBank:AAW82336.1
mRNA	DTHFPICIFCCGCCKTPKCG**L**CC**K**T	GenBank:AAV40979.1
mRNA	DTHFPICIFCCGCCKTPKCG**L**CC**K**T	NCBI RefSeq:AAV40979.1
recombinant peptide	DTHFPICIFCCGCCKTPKCG**L**CC**K**T	Peptides International
DNA	DTHFPICIFCCGCCKTPKCG**F**CC**R**T	This study
mRNA	DTHFPICIFCCGCCKTPKCG**F**CC**R**T	This study
peptide	DTHFPICIFCCGCCKTPKCG**F**CC**R**T	This study (PXD015661)

Previous accounts of the canine hepcidin peptide sequence determined by translation from a reported cDNA sequence are inconsistent with the hepcidin sequence when translated from independent (previously non-annotated) functional measurements including of the peptide isolated from *Canis lupis familiaris* and measured by mass spectrometry.

Equipped with the amended amino acid sequence of the dog hepcidin, a database search (NCBI) for other functional measurements was executed. The search revealed another mRNA measurement, submitted without annotation as a nucleic acid arising from dog liver, where *Hamp* expression is highest^36,37^, and of the tissue from where expression was first described^38^. This mRNA was also consistent with these described peptidogenomics identifications of the dog hepcidin and served as-yet another functional validation to that of our measurements (Table 1).

### Hepcidin-regulated serum iron concentration in the greyhound

**Figure 5:**
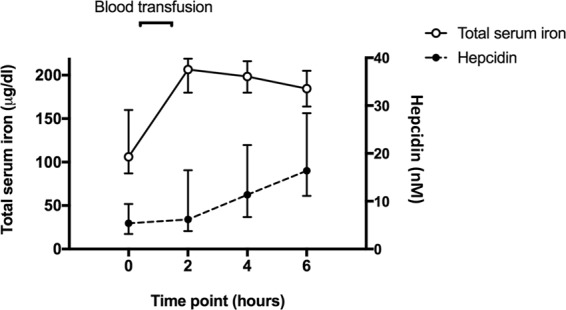
Median (Q1-Q3) of total serum iron and hepcidin peptide concentration (units, nM) in *Canis lupis familiaris*. Measurements were obtained from *Canis lupis familiaris* (greyhound; n = 10) measured at baseline (0 hours) and then 2 hours (after transfusion), 4 hours and 6 hours later. Change over time for both variables were significant at p < 0.05. Both total serum iron (p = 0.014) and hepcidin (p = 0.025) concentration increased significantly over time (Friedman’s test).

In confirming this first-described measurement of the *C. lupis familiaris* hepcidin peptide, we sought to confirm its physiological role as an iron homeostatic regulator within the dog. An assessment of iron metabolic diagnostics was undertaken in a greyhound hemorrhagic shock model following transfusion with iron-rich stored red blood cells, and subsequent analysis by isolation of peptides from the blood serum, followed by analysis of the hepcidin by LC-MS. Analysis of an added recombinant peptide standard (Peptides Internaional) determined good peak area reproducibility across the sample sequence of 117 (%RSD = 13.994), and all endogenous hepcidin measurements within the dynamic range of the standard curve. Physiologically, in agreement with expectations, an immediate and significant increase from baseline in total serum iron concentration was observed (p = 0.014) and followed more gradually by a significant increase in the identified *C. lupis familiaris* hepcidin concentration over time (p = 0.025; Fig. 5). The gradual increase in serum hepcidin from 2 hrs, coincided with a gradual reduction of total serum iron, consistent with a homeostatic response regulated by the hepcidin. As anticipated, the data are indicative of hepcidin upregulation in the presence of excessive iron to fulfil its physiological role in maintaining iron homeostasis.

### The evolutionary conservation of the Canidae hepcidin

**Figure 6:**
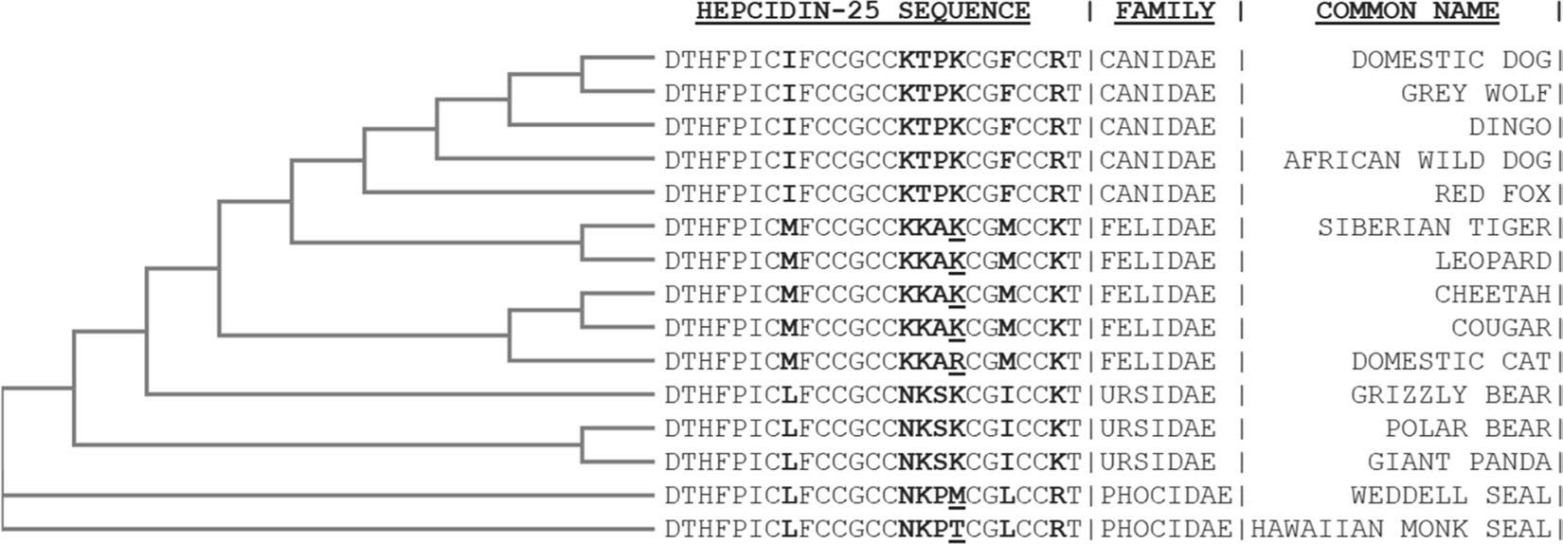
Multiple sequence alignment (ClustalW) identified the hepcidin peptide to be highly conserved between phylogenetic Families of the Carnivora Order. Perfect sequence identity was observed in the amino acids between members of the Canidae (*Canis lupis familiaris*, domestic dog, PRIDE_PXD015661; *C. lupis lupis*, Grey Wolf; *C. lupis dingo*, Dingo; Lycaon pictus, African Wild Dog CM007565.1; Vulpes vulpes, Red Fox, XM_026010384.1/XM_025989465.1) and within the Ursidae (*Ursus arctos horribillis*, Grizzly Bear, NW_020656198.1; *U. maritimus*, Polar Bear, NW_007930021.1; *Ailuropoda melanoleuca*, Giant Panda, XM_002920879.3). Whilst a single residue differed (underlined) between members within the Felidae (*Panthera tigris altaica*, Siberian tiger, XM_007096768.2; *P. pardus, Leopard, XM_019423254.1*; *Acinonyx jubatus*, Cheetah, XM_027044853.1; *Puma concolor*, Cougar, XM_025914594.1; *Felis catus*, domestic cat, XM_003997921.2) and Phocidae (*Leptonychotes weddellii*, Weddell Seal, XM_006730744.1; *Neomonachus schnauinslandi*, Hawaiian monk seal, XM_021700909.1). Individual amino acids which differ within the Carnivora are displayed in bold.

The evolutionary conservation of the *C. lupinus familiaris* hepcidin was next considered. Canines represent a diversity of evolution, not the least of which is represented in the domestic dog^39^. The conserved exons of the *Hamp* gene from three Canine species; *C. lupinus familiaris* (dog), *C. lupis lupis* (wolf) and *C. lupis dingo* (dingo) were aligned. The dog genome assembly (GCF_000002285.3) was complemented by the more recent wolf^40^ and dingo assemblies (GCF_003254725.1^41^, each of which agreed with the reverse-genetics validations and peptidogenomics result. Each compared ortholog of the Canine *Hamp* gene aligned with 100% sequence identity. Reverse-translation of the newly determined hepcidin sequence from the dog, reported here, also returned a perfect alignment score to the genome annotations. As such, we’re presented with evidence that the perfectly conserved amino acid sequence of this iron regulatory hormone has persisted through the diversity of evolutionary mechanisms applied to *C. lupinus familiaris* and the Canines more broadly. Further, we searched for evidence of conservation beyond the Canines. Predictions from the genome of the red fox (*Vulpes vulpes*) and African wild dog (*Lycaon pictus*) were consistent with our described *C. lupus familiaris* hepcidin, and broadened the characterisation to the Canidae family. Previous reports of the *Hamp* sequence determined by cloning and sequencing a *C. lupis familiaris Hamp* cDNA categorised its evolutionary conservation as that of the “canine hepcidin”^26^. This level of ontology implied sequence conservation with the *Canini* (canine) genera, but not of the broader Canidae family from which the Canini originate and which also includes the Vulpes (Red Fox). Speciation between the Canidae and Felidae however, show diversion of the predicted amino acid sequence of hepcidin, revealing the measurements obtained here to represent the hepcidin (HAMP) of genera within the Canidae family, but not of the Felidae (Fig. 6).

## Discussion

Hepcidin-25 is a key regulator of iron homeostasis in mammals. High concentrations of systemic hepcidin will reduce iron availability, by inactivation of the iron transporter ferroportin^3^ and resultant restriction of iron absorption and promotion of iron sequestration; including during inflammatory conditions^8^. As a biomarker, chronic deficiencies of hepcidin are associated with iron overload, and excessive hepcidin consistent with anaemias^10^. However, comparison of the presently accepted model of iron regulation, by measurement of the functional hepcidin peptide in animals beyond humans and mice are lacking in the literature. To establish greater mechanistic insight of iron homeostasis for translation from animal models, the availability of descriptive biomarkers such as hepcidin is important. Dogs being evolutionary carnivorous, and with a metabolic rate to body mass ratio^17^, and iron stores^22^ more representative to that of humans, than of mice^21–23^, could be considered to have a generally closer matched iron economy to that of humans.

Hepcidins are an evolutionarily conserved class of liver-expressed peptide. Here we have observed the *C. lupinus familiaris* hepcidin peptide to be perfectly conserved amongst the Canidae family of the Carnivora; broadening the previous definition of the amino acid sequence representing that of the Canines^15^. Whilst functionally it appears hepcidin is also highly conserved amongst mammals more broadly, the evolution of genomes has produced orthologous peptides amongst species, with subtle differences in amino acid sequence and mass. To permit specificity in measurement (if at all), the characterisation of hepcidin peptides for the given species under investigation should be undertaken. In this presented study, we used mass spectrometry to reveal the amino acid sequence identity of hepcidin in the dog, and exploited the high sequence conservation of hepcidin to challenge previous assumptions of the size of the molecule in the dog and in the greater Canidae. Complementary data resources of the genome and transcriptome of Canidae species and breeds thereof each provided a wealth of measurements for *in silico* prediction. Whilst typically, a peptidogenomics approach uses functional peptide measurement to curate genome annotations^42^, here rather, measurement of the functional peptide has been complemented by highly curated genome predictions, additionally serving to invalidate other functional measurements (in the form of incorrectly annotated mRNA transcripts and their translation). Indeed the only translations of the Canidae hepcidin which were consistent with the measured form within dog were identified in non-curated, or automated computed curations, of the nucleic acid sequence, rather than of (assumed) human-determined translations. Each of our independently determined genomic and previously non-annotated mRNA translations however were in agreement with the existence a Canidae hepcidin peptide of 2,777 Dalton, providing validation that our described identity and mass measurements of the Canidae hepcidin are indeed the Canidae ortholog of this hormone. With this determined discrepancy between the previously reported sequence and corresponding mass of the peptide, targeted MS methodology using a tandem MS instrument would not have resolved the Canidae hepcidin for measurement.

Further, the assumptions surrounding the iron regulatory role of the measured Canidae hepcidin hormone in dogs were tested using a greyhound model of hemorhagic shock^43^. The induction of an acute haemorrhage resulting in iron depletion has been shown to reduce hepcidin to baseline, ensuring maximum physiological availability of iron toward erythropoiesis^44^. Here, greyhounds subjected to a significant reduction of blood volume (inducing hemorhagic shock) were quickly transfused with iron-rich stored red blood cells, resulting in a rapid rise from baseline in total serum iron, and as expected an increase in serum hepcidin (Fig. 5). As a regulator iron of homeostasis, the anticipated response of hepcidin under such conditions of excess iron is to reduce the availability of iron by degradation of ferroportin. The observed increase in hepcidin from baseline concentrations at 2 hr post-transfusion (coinciding with excess serum iron), increasing significantly (p = 0.025) over the 6 hr experimental period was consistent with this anticipated response. Further, the gradual reduction of total serum iron following the initial rapid (post transfusion) increase, whilst serum hepcidin steadily rose, is consistent with hepcidin performing its role in iron homeostasis and bringing the physiological availability of iron back to a steady state. To this end, the isolation and quantification of this described peptide, the *Canidae* hepcidin, in the greyhound served as final translational validation to these described molecular characterisations. These data showed consistent agreement between the qualitative and quantitative assessment of the identification of this peptide being of the Canidae hepcidin, and the master iron regulatory hormone of these animals.

Together, these findings determine the dog (*C. lupis familiaris*) hepcidin to indeed be consistent with the evolutionary conservation of the greater Canidae, and in this determination, we provide an integral step toward the ready availability of this biomarker to translational studies employing dog models, and toward the establishment of hepcidin as a diagnostic marker in veterinary medicine.

## Methods

### Blood collection

Blood samples were obtained in a randomised controlled trial of dogs (*Canis lupis familiaris;* greyhound) transfused with stored red blood cells. Ten healthy ex-racing greyhounds were anaesthetized and a large bore catheter was placed in the femoral or carotid artery. Nine hundred mL of blood was removed from the arterial catheter over a period of 15 minutes. Immediately after withdrawal of blood, 15 mL.kg^−1^ of 5–6 week old packed red blood cells was administered intravenously over a period of 20 minutes. Blood samples were collected at baseline (prior to transfusion), and then 2 hours, 4 hours, and 6 hours after baseline. Serum was collected and stored at −80 °C in polypropylene cryo-tubes until analysis. For qualitative analysis, additional blood sera were later collected, consisting of serum from *Canis lupis familiaris* breeds*;* kelpie X, giant schnauzer, cavalier King Charles spaniel, Labrador retriever X and Great Dane.

### Total serum iron

The total serum iron of greyhound serum was assessed by first inducing ferric iron release from transferrin by introducing a strong acid, secondly reducing it to its ferrous form using ascorbate, and then complexing ferrous to a chromogen to induce a colour change quantified using spectrophotometry. Change over time for each variable was tested with the Friedman test (SAS Studio v3.91).

### Isolation and analysis of hepcidin by liquid chromatography mass spectrometry

Peptides were isolated from *C. lupis familiaris* blood serum and measured by Ultra Performance Liquid Chromatography quadrupole time-of-flight Mass Spectrometry (UPLC-qTOF-MS), as a modification of previously described methodology^45–48^, using a 5600 TripleTOF q-TOF-MS (Sciex, Massachusetts USA). For the preparation of serum, samples were thawed at room temperature for 90 min and subsequently mixed by vortex. A pooled sample was prepared by combining a 50 µl aliquot of each serum into a single polypropylene tube, which was mixed and dispensed into 400 µl replicate volumes. For quantitation, calibration samples were prepared from the pooled replicates by the addition of a recombinant hepcidin peptide (H-Asp-Thr-His-Phe-Pro-Ile-Cys-Ile-Phe-Cys-Cys-Gly-Cys-Cys-Lys-Thr-Pro-Lys-Cys-Gly-Leu-Cys-Cys-Lys-Thr-OH; Product PLP-3785-PI, Peptides International, Kentucky USA) dissolved in 40%v/v acetonitrile (ACN), in a final concentration range of 0.6–75 nM; representing an 8-point calibration. To account for changes in sample preparative conditions and/or instrument performance the same peptide standard was added to each dog sera in 400 µL volumes, to a final concentration of 18.75 nM. All calibration and experimental samples were randomised and prepared in a single batch. Larger polypeptides were precipitated by the addition of 800 µL of ACN whilst gently mixing the sample by vortex, followed by incubation at room temperature for 20 min. Precipitate was collected by centrifugation at 10,000* g* for 20 min and the supernatant was transferred to a fresh 2 mL polypropylene tube, frozen by submersion in liquid nitrogen and lyophylised to dryness in a Labconco Freezone 2.5 Freeze-dryer (Labconco, Missouri USA). Additional sample clean-up was by solid phase extraction using C18 silica (Prevail, Grace Discovery Science), performed by dissolving, binding to the sorbent and washing the peptide lyophilisate in 20%v/v ACN, with subsequent elution in 60% ACN. For LC-MS analysis the samples were again randomised, transferred to a glass analytical vial by pipette, and the eluent diluted to 20% ACN by the addition of water. Samples were injected into the chromatograph in 10 µl volumes. Chromatography was by reversed phase separation performed using a Shimadzu Nexera FPLC comprised of a DGU-20A5 degasser, LC-30AD pump, SIL-30AC auto-sampler, and a CTO-20A column heater (Shimadzu, Japan) coupled with an Aeris WIDEPORE 3.6 µ XB C18 column (2.1 × 100 m, Phenomenex Inc., USA) operated at a column temperature of 50 °C at a constant flow rate of 500 µL.min^−1^. Mobile phase A was prepared using LCMS grade water (0.2% v/v formic acid), solvent B as LCMS grade acetonitrile (0.2% v/v formic acid). All solvents were degassed for 15 minutes after preparation using a Digital Benchtop Ultrasonic Cleaner water bath (Soniclean, Australia). The chromatographic system was equilibrated at 10% solvent B, and the binary gradient ramped to 40% B over 5 minutes, and again to 99% B at minute 6, before re-equilibration at 10% B for each subsequent sample. The ion source was operated in positive electrospray ionisation (ESI) mode with an ion spray voltage of 5,500, declustering potential of 80, and at a temperature of 550 °C. The ion source nebuliser, heater and curtain gases (N_2_) were operated at 50, 45 and 30 psi, respectively. Data were acquired between m/z 50 and 1,000 in high resolution, with an accumulation of 0.15 sec. The MS was acquired with a background collision energy of 5 eV. The (M + 5 H)^+5^ ion of the putative hepcidin peptide observed in the TOF-MS spectra of *C. lupis familiaris* peptide isolates were targeted for subsequent MS/MS using two collision energies, 20 and 40 eV. For MS/MS experiments, the quadrupole was operated at a resolution of 0.7 Da on the precursor ion m/z 556.6 (M + 5H)^+5^, with an accumulation of 0.30 sec. MS data were collected at an operating resolution of ~30,000 for MS and MS/MS data. LC/MS and MS/MS spectra were acquired and interrogated using Analyst TF 1.7.1 and PeakView version 1.2.0.3 softwares (Sciex, Massachusetts USA).

### Data processing and peptide quantitation

Raw spectral data were processed using MarkerView version 1.2.1.1 (Sciex, Massachusetts USA). From the TOFMS trace, peak integration was using a noise threshold of 3 and minimum spectral width of 0.05 Da; with peaks aligned using a retention time tolerance of 0.10 min and mass tolerance of 0.05 Da. The peak area of the recombinant peptide internal standard was assessed for analytical reproducibility by calculation of the percent relative standard deviation (%RSD). For quantitation, the peak area of the endogenous hepcidin peptide was compared against the calibration sample series.

### Statistical analyses

Concentrations of serum iron and hepcidin from the greyhound haemorrhagic shock model were assessed for normality at each time point by visualisation of histograms and Q-Q plots. Within-group change over time for both iron and hepcidin concentration was tested using the Friedman test (SAS Studio v3.91), with a significance level set at *p* < 0.05. Given the aim was to report on overall trends of concentration over time, post-hoc pairwise comparison tests were not performed.

### Characterisation of the *C. lupis familiaris* peptide mass

Using the Institute for Systems Biology Proteomics Toolkit, Composition Calculator and MS/MS Fragment Ion Calculator (http://db.systemsbiology.net:8080/proteomicsToolkit/), the chemical composition of the putative hepcidin peptide was determined, following *de novo* elucidation of the amino acid sequence from the product ion (MS/MS) spectra. The mass was calculated with 8 hydrogen atoms removed to account for the post-translation modification of 4 disulfide bridges (consistent with the human hepcidin-25^49,50^), C_117_H_172_N_32_O_31_S_8_, and generating a monoisotopic mass prediction of 2777.06218 Da. Additionally, the monoisotopic mass of the serum-isolated hepcidin peptide, using the three observed charge states [M + 5H]^+5^ (m/z 556.420376), [M + 4H]^+4^ (m/z 695.273485) and [M + 3H]^+3^ (m/z 926.695333) was also determined, and found consistent with the ionisation of a 2777 Da peptide. The amino acid sequence was next entered into the Fragment Ion Calculator and following subtraction of a proton (−1.00794 Da) at each cysteine amino acid residue, a product ion mass spectrum was generated, calculated from an assumed [M + H^+^] charge state precursor, where M = 2777.06218 Da. The recombinant peptide standard (https://www.pepnet.com/Products/Detail/1863/Hepcidin-(canine)), although consistent with the mass of the published HAMP sequence, was significantly smaller than the mass of the putative hepcidin isolated from *C. lupis familiaris* serum.

### Database search for reported canine hepcidin sequences

The NCBI Reference Sequence database was searched for nucleic acid (mRNA) entries of the canine hepcidin antimicrobial peptide (*Hamp*). The search returned the originally described sequence NM_001007140.1^26^, and which is the reference sequence cited as the translation for the commercially available recombinant “Canine Hepcidin-25” peptide standard (Peptides International, Kentucky USA). This sequence was Basic Local Alignment Search Tool (BLAST) searched against the *Canis lupus familiaris* genomic sequence^51^. The search returned a nucleotide sequence match of 117/119 (98%) in Range 1, 107/109 (98%) in Range 2 and 63/63 (100%) in Range 3 to the RefSeq CanFam3.1, Boxer breed sequence, chromosome 1 (NC_006583.3). Protein BLASTp (protein to protein) of the same amino acid translation^26^ also matched, and identified other sequences NP_001007141.1^34^ and AY772532.1^33^; each of which cite the same original authorship^26^. To investigate the imperfect alignment between these previously annotated mRNA translations and the *Canis lupis familiaris* genomic sequence, the predicted *Hamp* gene region was translated directly from the heavily curated genomic sequence of *C. lupis familiaris*^51^ using ExPASy nucleotide to protein sequence translation tool (https://web.expasy.org/translate/). As anticipated, translation from the genome predicts a discrepancy with both the previous literature and database accounts of the sequence^26^. Translation from the *C. lupis familiaris* genome identified; range 1 to contain two nucleotide differences, which translate to an amino acid sequence; DTHFPICIFCCGCCKTPKCGFCCRT, with previously unreported phenylalanine and arginine amino acids at the 21^st^ and 24^th^ positions (Table 1).

### Identification of Non-annotated *C. lupis familiaris* Hepcidin mRNAs

To establish if the originally reported amino acid sequence of canine hepcidin^26^ were indeed incorrectly annotated, Tblastn (Protein to translated nucleotide) of the genomic-translation against the expressed sequence tag (est) database was executed and returned a *C. lupis familiaris* (beagle) liver mRNA sequence^36^ with a perfect alignment to the prediction. Finally, the predicted sequence were also searched using Ensembl Genebuild entry, generated using automated annotation from CanFam 2.0^52^, which also returned an amino acid sequence with perfect alignment to the genomic *Hamp* prediction, though inconsistent with the earlier published sequence^26^.

### Comparison of the predicted canine hepcidin to other *Canidae* species

The BLAST workflow was repeated for the recently published dingo genome^53^. The predicted hepcidin nucleotide sequence from CanFam3.1 was BLAST Genome searched against the dingo genome. The gene regions accessions aligned with 100% sequence identity to the 3 matched ranges of *Canis lupus dingo* isolate Sandy unplaced genomic scaffold, ASM325472v1 SuperScaffold_32 (NW_020269188.1). The grey wolf genome is a recent release^40^ and not yet available for BLAST search. As such the FASTA sequence file for the entire genome was downloaded and viewed using Large Text File Viewer (Version 5.2 u). The 5′-3′ DNA nucleotide sequence (*Hamp*) which translates to the HAMP sequence DTHFPIC (reverse translation; ATGCAGATGGGGAAGTGGGTGTC), and is highly conserved amongst mammals were searched and identified in Scaffold_31 at text line 2,657,851 (of 28,924,874). Within the surrounding sequence the same 3 gene ranges from dog and dingo were identified and found to match with 100% sequence identity.

### Multiple sequence alignment (ClustalW)

To observe the evolutionary relationship between the determined peptide sequences, the amino acid residues were aligned by ClustalW using Clustal Omega Multiple Sequence Alignment^54^.

### Data sharing statement

All genomics and transcriptomics data are freely available in the aforementioned databases and can be found using the referenced accession codes. All additional data supporting these described findings are provided within the paper and its supplementary information, and raw mass spectrometry data available through the Proteomics IDEntifications (PRIDE) database, Project PXD015661 (ebi.ac.uk/pride/archive).

### Ethics statement

This study was approved by the Murdoch University Animal Ethics Committee (R2732/15 and NC2836/16 (510) and was conducted in accordance with the Australian Code for the Care and Use of Animals for Scientific Purposes.

## Supplementary information

Supplementary information
