# Supplementary material for: Identification of The Canidae Iron Regulatory Hormone Hepcidin
Source: Sci Rep. 2019 Dec 18;9:19400. doi: 10.1038/s41598-019-55009-w (PMC6920140; doi:10.1038/s41598-019-55009-w)
Supplement: Supplementary file 1 — Supplementary information [file 41598_2019_55009_MOESM1_ESM.pdf]

IDENTIFICATION OF THE CANIDAE IRON REGULATORY HORMONE HEPCIDIN

Martin K. Mead[1], Melissa Claus[2], Edward Litton[3,4], Lisa Smart[2], Anthea Raisis[2], Gabriele Rossi[2], Robert D. Trengove[5,6], Joel P. A. Gummer*[5,6].

[1]School of Veterinary and Life Science, Murdoch University, WA, Australia
[2]School of Veterinary Medicine, Murdoch University, WA, Australia
[3]Intensive Care Unit, Fiona Stanley Hospital, WA, Australia
[4]School of Medicine, University of Western Australia, WA, Australia
[5]Metabolomics Australia, Western Australia Node, Murdoch University, WA, Australia
[6]Health Futures Institute, Murdoch University, WA, Australia.

*To whom correspondence should be addressed:
Joel P.A. Gummer, j.gummer@murdoch.edu.au
Australian National Phenome Centre (ANPC), Harry Perkins Institute for Biomedical Sciences,
5 Robert Warren Drive, Murdoch Western Australia, Australia 6150 | Tel/Fax +618 9360 6000

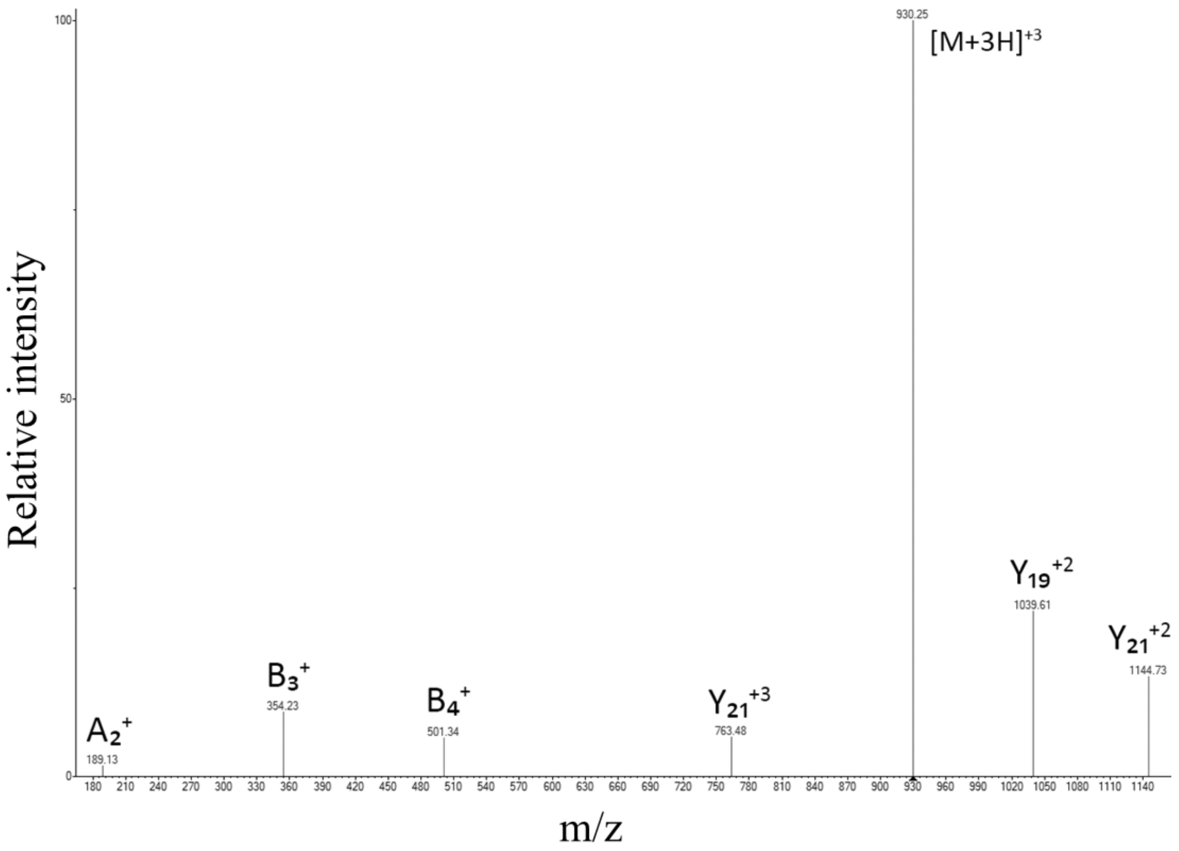

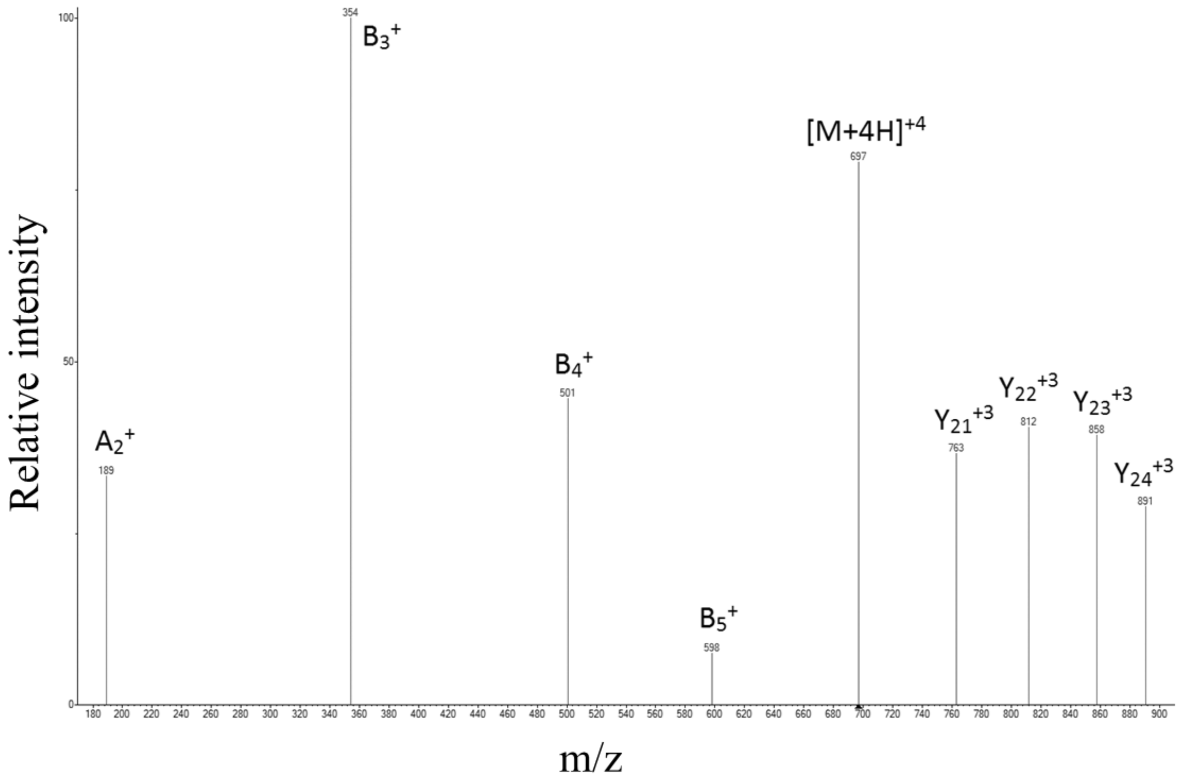

Supporting (S1). Product ion mass spectra of the human hepcidin-25, generated by dissociation of the $[M+3H]^{3+}$ (Upper) and $[M+4H]^{4+}$ (Lower) precursor ions, measured by mass spectrometry using electrospray ionisation (ESI). Amino acid sequence conservation of the hepcidin peptide amongst species predicted the b-series ions to also be consistent amongst species.

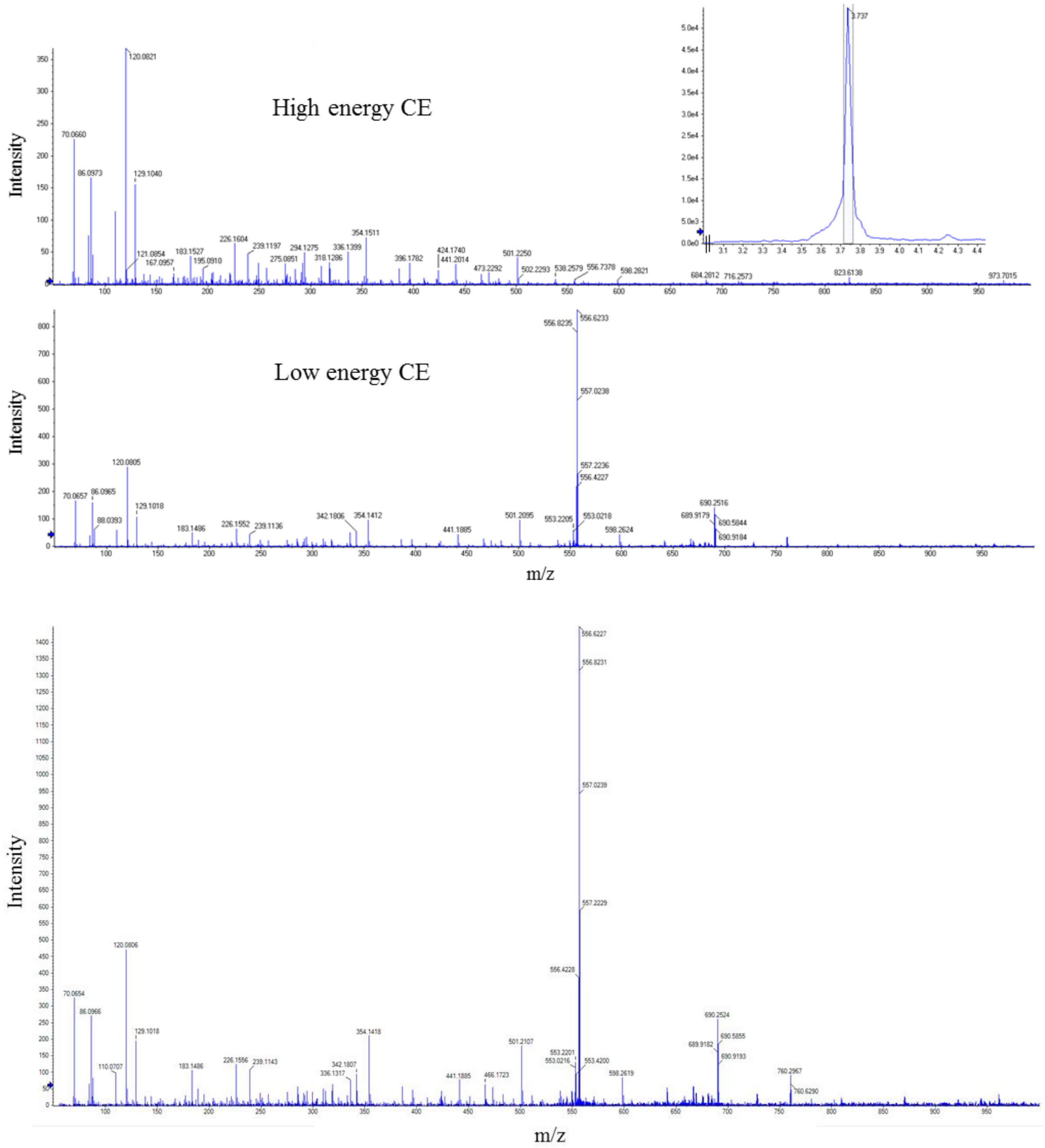

Supporting (S2): Raw product ion mass spectrum (MS/MS) used in the confirmation of the peptide as a hepcidin-25, and determination of the amino acid sequence of the confirmed Canidae hepcidin. The total ion chromatogram of the hepcidin peak from the MS/MS trace is also pictured (top right).

Supporting (S3): Cited canine hepcidin-25 nucleic acid sequences.

Type	Determined Canine Hepcidin-25 Nucleic Acid Sequences (5'-3')	Source/Database Identifier	Study Author
mRNA	gacacccacttccccatctgcatattctgctgtggctgc tgtaaaacaccgaagtgtgggctctgctgcataaca	GenBank: AY590589.1	[1]
mRNA	gacacccacttccccatctgcatattctgctgtggctgc tgtaaaacaccgaagtgtgggttgtgctgcaagacg	GenBank: AY772532.1	[2]
mRNA	gacacccacttccccatctgcatattctgctgtggctgc tgtaaaacaccgaagtgtgggttctgctgcaggacg	GenBank: CO675791.1	[3]
mRNA	gacacccacttccccatctgcatattctgctgtggctgc tgtaaaacaccgaagtgtgggttctgctgcaagacg	GenBank: AY899807.1	[4]
DNA	gacacccacttccccatctgcatattctgctgtggctgc tgtaaaacaccgaagtgtgggttctgctgcaggacg	Ensembl: ENSCAFT00000011304	[5]
mRNA	gacacccacttccccatctgcatattctgctgtggctgc tgtaaaacaccgaagtgtgggttgtgctgcaagacg	NCBI Reference Sequence: NM_001007140.1	[6] (2005-2017 Update)
Recombinant peptide	gacacccacttccccatctgcatattctgctgtggctgc tgtaaaacaccgaagtgtgggttgtgctgcaagacg	Product Code: PLP-3785-PI (reverse translation)	[7]
Peptide	gacacccacttccccatctgcatattctgctgtggctgc tgtaaaacaccgaagtgtgggttctgctgcaggacg	Reverse translation of [30, 35]	This Study
